# The Impact of Rendered Protein Meal Oxidation Level on Shelf-Life, Sensory Characteristics, and Acceptability in Extruded Pet Food

**DOI:** 10.3390/ani6080044

**Published:** 2016-07-28

**Authors:** Sirichat Chanadang, Kadri Koppel, Greg Aldrich

**Affiliations:** 1 Sensory Analysis Center, Department of Food Nutrition Dietetics and Health, Kansas State University, 1310 Research Park Drive, Manhattan, KS 66502, USA; sirichat@ksu.edu; 2 Department of Grain Science and Industry, Kansas State University, Manhattan, KS 66506, USA; aldrich4@ksu.edu

**Keywords:** aroma, chicken byproduct meal, beef meat and bone meal, rancidity

## Abstract

**Simple Summary:**

Sensory analysis was used to determine the changes due to the storage time on extruded pet food prepared from two different rendered protein meals: (i) beef meat and bone meal (BMBM); (ii) chicken byproduct meal (CPBM). Extrusion is a process where feed is pressed through a die in order to create shapes and increase digestibility. Descriptive sensory analysis using a human panel found an increase in undesirable sensory attributes (e.g., oxidized oil, rancid) in extruded pet food over storage time, especially the one prepared from chicken by product meal without antioxidants. The small increase in oxidized and rancid aromas of BMBM samples did not affect pet owners’ acceptability of the products. CPBM samples without antioxidants showed a notable increase in oxidized and rancid aroma over storage time and, thus, affected product acceptability negatively. This finding indicated that human sensory analysis can be used as a tool to track the changes of pet food characteristics due to storage, as well as estimate the shelf-life of the products.

**Abstract:**

Pet foods are expected to have a shelf-life for 12 months or more. Sensory analysis can be used to determine changes in products and to estimate products’ shelf-life. The objectives of this study were to (1) investigate how increasing levels of oxidation in rendered protein meals used to produce extruded pet food affected the sensory properties and (2) determine the effect of shelf-life on pet owners’ acceptability of extruded pet food diet formulated without the use of preservative. Pet food diets contained beef meat bone meal (BMBM) and chicken byproduct meal (CBPM) in which the oxidation was retarded with ethoxyquin, mixed tocopherols, or none at all, and then extruded into dry pet foods. These samples represented low, medium, and high oxidation levels, respectively. Samples were stored for 0, 3, 6, 9, and 12 months at ambient temperature. Each time point, samples were evaluated by six highly trained descriptive panelists for sensory attributes related to oxidation. Samples without preservatives were chosen for the acceptability test, since the differences in sensory characteristics over storage time were more distinguishable in those samples. Pet owners evaluated samples for aroma, appearance and overall liking. Descriptive sensory analysis detected significant changes in oxidized-related sensory characteristics over storage time. However, the differences for CBPM samples were more pronounced and directional. The consumer study showed no differences in pet owners’ acceptability for BMBM samples. However, the noticeable increase in aroma characteristics (rancid aroma 0.33–4.21) in CBPM samples over storage time did have a negative effect on consumer’s liking (overall liking 5.52–4.95).

## 1. Introduction

The pet supply industry is an important part of the U.S. economy with total expenditures in 2015 of $60.59 billion and $23.04 billion of that spent on food [1]. Rendered protein meals such as poultry byproduct meal, meat, and bone meal, and fish meal are widely used in pet food industry [2]. These rendered products are generally an excellent source of protein, energy and minerals [2,3]. Pet foods are expected to maintain their desired characteristics for 12 months or more. Holding a food for this extended period of time is difficult. Therefore, antioxidants become an important addition to pet foods to prevent the deterioration through oxidation of lipids and preserve nutrient quality [4]. Lipid oxidation is the major factor that causes off-odors, off-flavors, possibly produces toxic compounds, and affects the shelf life of food products, including pet food [4,5]. The most effective antioxidants are the synthetic preservatives such as ethoxyquin, butylated hydroxyanisole (BHA), and butylated hydroxytoluene (BHT) [4]. These preservatives are added to the raw ingredients at the time of production, and then again during the pet food production processes, to assure the food is produced from “fresh” ingredients and then once produced has enough residual preservatives to hold the food until consumed by the pet many months later. Natural antioxidants such as mixed tocopherols can also be effectively used as preservatives in pet foods. However, the natural preservatives are not as effective as the synthetic ones [6]. In this study, the synthetic antioxidant ethoxyquin was used to create a “low” oxidation level treatment, while mixed tocopherols were used to manufacture a “medium” oxidation level, and then no antioxidants were added to the samples representing “high” oxidation level. 

There are several methods that are commonly used to measure lipid oxidation in pet food industry such as peroxide value (PV), oxygen consumption (oxygen-bomb), thiobarbituric acid reactive substances (TBARS), and gas chromatography-mass spectrometry (GC-MS) for the quantitative analysis of volatile compounds such as aldehydes and ketones [7]. Relating an acceptable level of lipid oxidation measured from those analytical methods is not well defined. Sensory analysis is another method that can be used to determine the level of lipid oxidation and shelf life of food products. Shelf lives of many food products are limited by the changes in their sensory characteristics [8]. Those changes can occur before any risk to consumers’ health is reached, especially foods that do not tend to suffer from microbiological changes such as baked goods and flour [9]. Descriptive sensory analysis using a human panel enables quantification of the aroma, flavor, appearance, and texture properties of food and non-food products, including pet food [10]. Rancidity-related sensory attributes, which are the indicator of lipid oxidation, have been detected and evaluated in pet foods [11,12,13]. Furthermore, when pet food is served to the pet, the owner is the mediator and an evaluator of the pet food acceptability. In case the pet food exhibits off-aromas that are related to an unacceptable product, the pet owner may elect not to serve those foods to their pet. Therefore, using sensory profiles created by human panel and acceptability data from pet owners may enable a more rapid, quantitative and predictive indication of the effects of ingredients and changes on the products due to processing and storage [11,13].

In this study the dry pet food samples using chicken byproduct meal or beef meat and bone meal were created to represent low, medium, and high oxidation levels through the use of ethoxyquin, mixed tocopherols, and no added antioxidants, respectively. The objectives of this study were to: (1) investigate how increasing levels of oxidation in rendered protein meals used to produce extruded pet food affected the sensory properties and (2) determine the effect of shelf-life on pet owners’ acceptability of extruded pet food diet formulated without the use of preservative.

## 2. Materials and Methods

### 2.1. Rendered Protein Meal

Rendered protein meal (approximately 454 kg of each) from beef meat and bone meal (BMBM) and chicken byproduct meal (CBPM) were acquired from cooperating rendering plants. Each meal had been ground and was split into three equal portions. The first portion of BMBM and CPBM were left untreated and labeled BMBM-O and CBBM-O. These samples represented the high oxidation level treatment. The second portion of BMBM and CPBM were preserved with a 1:10 wt:wt dilution of mixed tocopherols (Naturox™, Kemin Industries, Des Moines, IA, USA) and labeled BMBM-MT and CBPM-MT. These samples represented the medium oxidation level treatment. The last portion of BMBM and CBPM were preserved with a 1:10 wt:wt dilution of ethoxyquin (Rendox™, Kemin Industries, Des Moines, IA, USA) and labeled BMBM-ET and CBPM-ET. These samples represented the low oxidation level treatment. All samples were kept at room temperature (25 °C, 51% relative humidity). The BMBM samples were stored for 63 days and the CBPM samples for 41 days prior to extrusion.

### 2.2. Diet

The rendered protein meal was the primary protein source in the experimental diets along with the other ingredients (grains, fibers, vitamins, and minerals) sourced from supplies standard to pet food production. The diets were formulated to be iso-proteinaceous (30% crude protein level at 10% moisture). Beef meat and bone meal is often lower in protein and higher in ash than chicken by product meals and, therefore, required more protein meal [7]. All ingredients were individually weighed prior to mixing in a double ribbon mixer (Wenger Manufacturing, Sabetha, KS, USA) for three minutes before the micro ingredients were added and allowed to mix an additional three minutes. After mixing, the diets were then bagged in 22.7 kg paper bags in preparation for extrusion. All six experimental pet food diets (BMBM-O, BMBM-MT, BMBM-ET, CBPM-O, CBPM-MT, CBPM-ET) were produced at the BIVAP Extrusion Laboratory; Kansas State University; Manhattan, KS, USA (Table 1).

### 2.3. Extrusion Process

The dry mixed ingredients were extruded on a single screw extruder (Wenger X-20, Wenger Manufacturing; Sabetha, KS, USA) using a typical pet food screw profile. The extruder screw profile included 1-Inlet screw, 2-Single flight full-pitch screw, 3-Small shear lock, 4-Singleflight full-pitch screw, 5-Small shear lock, 6-Single flight screw, 7-Medium shear lock, 8-Doubleflight single pitch screw, 9-Large shear lock, 10-Double flight cut cone screw. The extruder barrel jacket temperature for zone one was 30 °C, zone two was 70 °C, and zone three was 90 °C. The extruder die shape and size was a 5 mm circle with a knife setup of six solid blades.

The target extruder conditions for moisture were 27%–30% and a dry feed rate of 180 kg/h. The steam was added at a rate of two-thirds the water in the pre-conditioner (discharge temperature 95.5 °C) while the remainder of the moisture was added to the extruder barrel and recalculated to achieve the processing moisture goal. The target bulk density was 350 g/L. The target extruder screw speed was 340 to 450 rpm based on the control diets performance. Throughput for BMBM treatments averaged 220 kg/h and CBPM treatments 204 kg/h. Once the parameters for the control diet were set, they were held constant for the remainder of the experimental treatment processing. 

The extruded kibble exiting the extruder was pneumatically conveyed to a dual pass dryer/single pass cooler (Wenger 4800 Series, Wenger Manufacturing; Sabetha, KS, USA). The dryer was set at 99 °C and 10 min per pass and 10 min through the cooler. The dryer conditions were adjusted to achieve a target final moisture of 7.5%. The pet food was not coated with flavors or fats upon exiting the dryer to eliminate confounding factors.

### 2.4. Shelf Life

Total of 3 kg of kibble per treatment were placed in freezer storage bags. Each bag was punctured with a pin sized hole to facilitate air exchange. Each bag was labeled with their respective treatment and storage duration. Samples were stored in a covered plastic tote at ambient condition (~22 °C and 45% relative humidity) for 0, 3, 6, 9, and 12 months. Pet food diets produced from BMBM and CBPM preserved with mixed tocopherols, ethoxyquin and unpreserved at all time points were pulled from the shelf-life test and stored in the freezer (−18 °C) until descriptive sensory analysis and consumer testing were performed.

### 2.5. Descriptive Sensory Analysis

Descriptive sensory analysis was conducted by six highly-trained panelists. They were trained according to guidelines provided in American Standard Test Method [14] and Meilgaard et al. [15]. Panelists received two 1.5 h orientation sessions on dry pet food using samples that were included in this study before proceeding with sensory tests in order to introduce the products. The appropriate sensory attributes selected from a lexicon developed for this product category [11] and technique for evaluating samples in this study was designed during these orientation sessions.

Evaluation of all samples was conducted during six 1.5 h sessions. Each test sample was served in a ~100 mL plastic cup for flavor evaluation, and in a medium snifter (4 g) covered with a watch glass for the evaluation of aroma attributes. Stale, oxidized oil, rancid, and cardboard aroma, and flavor were evaluated in all samples (Table 2). Furthermore, fundamental tastes including sour, bitter, and metallic were evaluated. 

For the evaluation, a numeric scale of 0–15 with 0.5 increments where 0 represents none and 15 extremely high was applied to each attribute to provide a measure of intensity. The samples were evaluated in duplicate in a randomized order. Similar evaluation methods have been used in the acceptability study for dry dog foods variations [16], and the studies on the effects cooking process and inclusions (meat and fiber) on pet food sensory characteristics [17,18]. Microbiological testing (i.e., absence of *Salmonella* and coliform bacteria) was conducted on all samples prior to sensory evaluation to ensure safety. All testing was completed under approval from Kansas State University Internal Review Board.

### 2.6. Consumer Acceptance

Acceptance of the experimental pet foods was tested using a Central Location Trial (CLT). Both foods produced with BMBM and CBPM samples without antioxidant (BMBM-O and CBPM-O) at 0, 3, 6, 9, and 12 months were selected for consumer study. These samples represented the “high” oxidation levels as no antioxidants had been added to these samples during manufacturing. A total of 10 samples were evaluated by each consumer. A total of 106 pet owners who feed their pets dry food were recruited from the consumer database at the Sensory Analysis Center (Manhattan, KS, USA). The pet owners were screened for dog or cat ownership, information about the breed, diet of the dog(s) and cat(s), and owner demographic information. The pet owners had to be willing to participate in this study and have no allergies. During the Central Location Trial, conducted at the Sensory Analysis Center, questionnaires were administered by RedJade software (RedJade^®^, Redwood Shores, CA, USA). Blind-coded samples were served to the pet owners monadically in a randomized order. Half of the pet owners evaluated CBPM samples in a randomized order first, then had a small break, and next evaluated the BMBM samples in a randomized order. The other 50% of the pet owners evaluated the BMBM samples first, followed by a break, and then evaluated CBPM samples. The pet owners were asked to evaluate their overall liking, appearance liking, and aroma liking on a nine-point hedonic scale where 1 indicated “dislike extremely”, and 9 indicated “like extremely”. Pet owners were also asked about aroma intensities on a five-point Just-About-Right (JAR) scale where 1 indicated “too weak”, 3 “just about right”, and 5 “too strong”. They were asked to describe their specific likes and dislikes for each sample using open-ended questions. In addition questions about their feeding behavior, dog/cat food storage, and money they spend on pet food were also included. The pet owners were compensated for their time. 

### 2.7. Data Analysis

Data from BMBM and CBPM diets were analyzed separately.

Descriptive sensory data for each diet were analyzed by repeated-measures analysis overtime using PROC GLIMMIX procedure (SAS version 9.4, The SAS Institute Inc., Cary, NC, USA). The fixed effects in the model included antioxidant treatment (mixed tocopherols, ethoxyquin, and unpreserved), storage time and their interaction. Replication and panelist were considered as random effects in the model. The statistical significance of differences for main effects and interactions were defined as *p* ≤ 0.05. Fishers protected Least Significant Difference (LSD) was used to determine significant effects of antioxidant treatment and storage time. 

Liking scores from consumer study were analyzed by a one-way ANOVA mixed effect model (SAS version 9.4, The SAS Institute Inc., Cary, NC, USA) using PROC GLIMMIX to determine significant differences among samples on liking scores. For all significant liking scores, the sample effects were assessed using pair-wise comparisons based on SAS least square (LS) means. The criteria for significance was *p* ≤ 0.05. 

Penalty analysis for just-about-right (JAR) attributes for all storage time points were performed using XLStat version 2015.3.01 (Addinsoft, New York, NY, USA). The *p*-value was calculated only if each level (too little, JAR, too little) had more than 20% consumers which was the typical cut-off criteria for this analysis.

Partial least square regression (PLSR) was used to create external preference mapping by regressing descriptive attributes and consumer liking data to explore the drivers of liking for dry pet food. PLSR was performed using XLStat version 2015.3.01 (Addinsoft, New York, NY, USA).

## 3. Results

### 3.1. Descriptive Sensory Analysis

#### 3.1.1. Beef Meat and Bone Meal (BMBM)

The mean intensity scores of 11 sensory characteristics for pet food prepared from BMBM are shown in Table 3 and Table 4.

The antioxidant treatment × storage time interaction was significant in all evaluated aroma attributes (*p* ≤ 0.05), indicating that antioxidant effect responded to storage time differently (Table 3). There were significant differences among storage times (*p* ≤ 0.05) in all aroma attributes, but antioxidant effect was seen only in oxidized oil and stale aroma. Within each treatment, the intensities of oxidized oil, stale, and rancid aroma significantly increased when the storage time increased. Interestingly, the sample preserved with mixed tocopherols (BMBM-MT) was the sample which had largest changes in oxidized oil and rancid attributes over time. The unpreserved sample (BMBM-O) was the one that seemed to have the smallest changes on those two aroma characteristics and no significant change in cardboard aroma. Within each storage time point, food preserved with ethoxyquin (BMBM-ET) had highest intensity (*p* ≤ 0.05) in stale aroma at the beginning of the study through six months of storage. BMBM-MT was significantly higher in oxidized oil aroma than the other treatments at 12 months (*p* ≤ 0.05).

The interaction effect was significant (*p* < 0.05) in four out of seven evaluated flavor attributes (Table 4). Oxidized oil, stale, and rancid flavors were significantly different across storage time (*p* ≤ 0.05), considering each antioxidant treatment separately. The BMBM-MT sample showed a significant increasing trend in oxidized oil and stale flavor over time. This was not clearly seen in the other two treatments. Rancid flavor significantly increased over time for BMBM-MT and BMBM-ET samples (*p* ≤ 0.05) but not for BMBM-O sample. Antioxidant treatment did not have significant effect (*p* > 0.05) on most attributes, except stale and metallic. BMBM-ET sample was significantly higher in stale intensity compared to the other treatments at three months (*p* ≤ 0.05). While BMBM-MT sample had the lowest intensity (*p* ≤ 0.05) in metallic at the beginning of the study through three months of storage, BMBM-ET sample had the lowest (*p* ≤ 0.05) in metallic intensity at 12 months of storage. These results indicate that different oxidation levels do not necessarily have the expected directional effect on the sensory properties of pet foods.

#### 3.1.2. Chicken Byproduct Meal (CBPM)

The mean intensity scores of 11 sensory characteristics for pet food prepared from CBPM are shown in Table 5 and Table 6.

The antioxidant treatment x storage time interaction was significant (*p* < 0.05) in oxidized oil and rancid aroma (Table 5). It appeared that storage time affected control sample (CBPM-O) differently from the other two treatments. Antioxidant treatment and storage time showed significant effects (*p* ≤ 0.05) on most of aroma attributes, except cardboard that had no significant differences across time point (*p* > 0.05). It was shown that the unpreserved sample (CBPM-O) had significantly increased (*p* ≤ 0.05) oxidized oil and rancid aroma when it had been stored for longer time. On the other hand, there were no significantly different (*p* > 0.05) oxidized oil and rancid aroma intensities when storage time increased for samples preserved with antioxidants (CBPM-MT and CBPM-ET). At the storage time of 12 months, the CBPM-O sample showed the highest intensity in all aroma characteristics (*p* ≤ 0.05).

The results reported in Table 6 were also in the same direction as results in Table 5. An interaction effect was seen only on oxidized oil and rancid flavor (*p* ≤ 0.05). Looking at each antioxidant treatment, all attributes except sour were significantly different across storage time (*p* ≤ 0.05). The intensity of oxidized oil flavor, stale flavor, rancid flavor, and metallic flavor significantly increased when storage time increased for the control sample (CBPM-O). There were also significant changes on oxidized oil, cardboard, rancid, bitter and metallic flavors between storage time for the samples preserved with antioxidants (CBPM-MT and CBPM-ET), however, the changes were minimal compared to the changes occurred in CBPM-O sample. Antioxidant treatment also had a significant effect on oxidized oil, stale, cardboard, and rancid flavors (*p* ≤ 0.05). The control sample (CBPM-O), which had been kept for 12 months, had the highest intensity on oxidized oil, stale, and rancid flavors (*p* ≤ 0.05). These results indicate that oxidation process is highly dependent on the ingredients used in pet foods and that oxidation levels may need to be managed using different strategies based on the ingredients used.

### 3.2. Consumer Acceptance

It was observed from descriptive analysis results that the differences for rendered protein meal pet food samples without antioxidants (high-oxidation level) over storage time were more pronounced and directional, especially for CBPM samples. Therefore, rendered protein meal samples (for both BMBM and CBPM) produced without antioxidants were chosen for the consumer study.

A total of 106 pet owners completed the study. The demographic information of participants in this study is shown in Table 7. The participants in this study either had dogs (58.4%), cats (20.8%), or both cats and dogs (20.8%). While dog owners had more purebred dogs than mixed breed, cat owners owned more mixed breed cats than purebred ones. Most pet owners stored their dry pet food in airtight containers (49.1%) or resealed in the original package (34.9%). The majority of them spent less than $100 on pet food per month and normally finished a package of dry pet food within one month (68.9%).

#### 3.2.1. Acceptability of Shelf-Life Samples

##### Beef Meat and Bone Meal (BMBM-O)

The mean scores for overall liking, appearance liking and aroma liking for BMBM-O samples are shown in Table 8. The results showed that storage time did not affect pet owners’ acceptance (overall, appearance, or aroma liking) on dry pet food prepared from unpreserved diet (BMBM-O) (*p* > 0.05). However, we noticed that the average liking scores for all samples were in the range of “slightly dislike—neither like nor dislike”. This meant pet owners were not particularly fond of these samples, even the fresh sample (0 month).

The majority of pet owners said the things that made them like this sample was the size, which was just about right for their pets. On the other hand, they did not like this sample because it looked too dry, had a bland color, and had a low intensity in an appetizing smell (e.g., meaty). In addition, a lot of them reported that they found hair and white pieces in the sample, which did not appeal to them.

##### Chicken Byproduct Meal (CBPM-O)

The acceptability results for CBPM-O samples from Table 8 showed that there were no significant differences between samples for appearance liking (*p* > 0.05). On the other hand, there was a significant difference between samples for overall liking and aroma liking (*p* ≤ 0.05). There was a significant decrease in overall liking and aroma liking scores for samples with a longer shelf-life. CBPM-O sample kept for 12 months had the lowest scores in term of the acceptability in overall and aroma.

The liking scores for the fresh samples (0 month) prepared from chicken byproduct meal (CBPM-O) were somewhat higher than the one prepared from beef meat and bone meal (BMBM-O). The liking scores for the fresh sample were in the range of “Like slightly—Neither like nor dislike”. The main reason for higher liking scores was no detection of hair or white pieces in the sample. However, some consumers still mentioned that they did not like the samples due to the unappealing color.

#### 3.2.2. Penalty Analysis

##### Beef Meat and Bone Meal (BMBM-O)

Penalty analysis was conducted to determine whether the consumers “penalized” the samples for having too high or too low aroma characteristics. Table 9 showed that there was a significant drop in aroma liking score at *p* ≤ 0.05 for the BMBM-O samples that had too low aroma. This means the aroma-liking score was decreased by 0.685 points when samples did not have enough aroma characteristics that were clearly perceivable by consumers.

##### Chicken Byproduct Meal (CBPM-O)

Contrary to BMBM-O samples, pet owners strongly penalized the CBPM-O samples which had too intense aromatics (*p* = 0.020). The aroma-liking score dropped by 1.166 points for those samples that had too strong aromatics (Table 9).

Based on the descriptive data from Table 5 and Table 6, the off-note characteristics (oxidized oil and rancid) were obviously increased when samples had been stored for 12 months. The high intensity of the off-note characteristics might have exceeded consumers’ acceptability and lead the consumer to consider these higher intensities as unpleasant aromas for the dry pet food. Therefore, the higher intensity of off-note characteristics resulted in the lower liking score of the samples.

#### 3.2.3. Drivers of Liking

##### Beef Meat and Bone Meal (BMBM-O)

**Figure 1:**
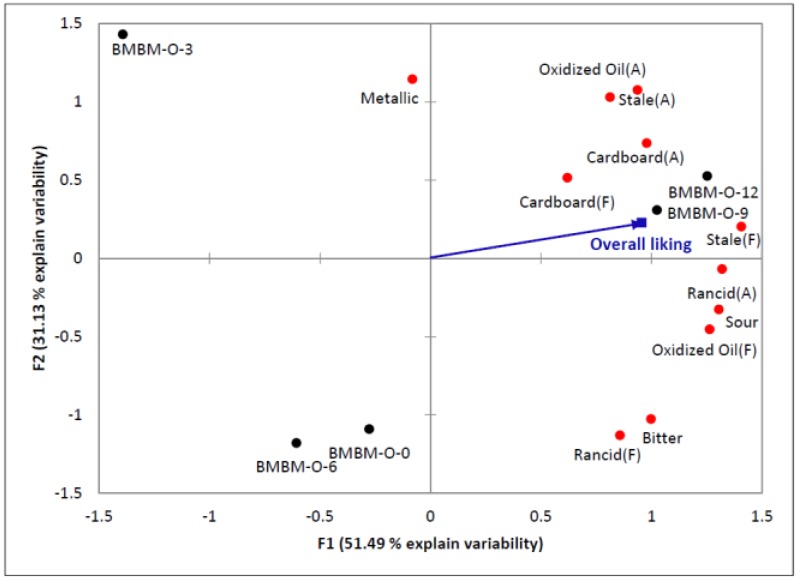
External preference mapping of BMBM-O samples from five different time points and average overall liking from 106 pet owners. Black dots represent samples from specific time points; red dots represent sensory attributes. The two dimensions accounted for 82.62% of total variability in the sensory data (F1 accounted for 51.49% and F2 accounted for 31.13%).

The external preference mapping in Figure 1 combined descriptive sensory analysis data with consumers’ overall liking scores for BMBM-O samples. Based on the preference map, the samples with higher intensities in off-note characteristics (samples kept for nine and 12 months) were preferred by pet owners. Pet owners gave lower liking scores for the fresh sample and the samples kept for 3 and 6 months due to the lower overall aroma intensity. However, there were no significant differences in liking scores across samples. This result agreed with the penalty analysis result. Higher intensity in off-note characteristics for BMBM samples kept for nine and 12 months might not have been strong enough for consumers to detect and, hence, was not considered as an undesirable aroma.

##### Chicken Byproduct Meal (CBPM-O)

**Figure 2:**
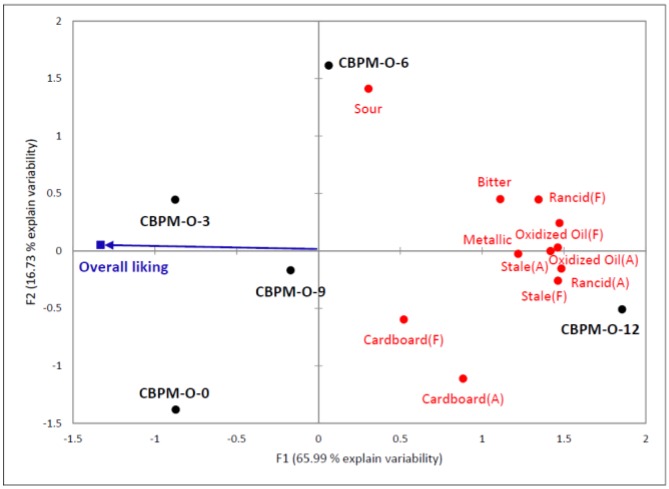
External preference mapping of CBPM-O samples from 5 different time points and average overall liking from 106 pet owners. Black dots represent samples from specific time point; red dots represent sensory attributes. The two dimensions accounted for 82.72% of total variability in the sensory data (F1 accounted for 65.99% and F2 accounted for 16.73%).

The external preference mapping in Figure 2 showed that CBPM-O samples with lower intensities in off-note characteristics seemed to be preferred by pet owners. The descriptive sensory data showed the dramatic increase in off-note characteristics, especially oxidized oil and rancid attributes (*p* ≤ 0.05). The increase in off-note intensity in samples kept for 12 months was high enough for consumers to detect the differences. Moreover, these intensities tended to exceed their acceptability and this resulted in significantly lower liking scores for the samples kept for 12 months.

## 4. Discussion

Descriptive sensory analysis showed that BMBM samples preserved with antioxidants (BMBM-ET and BMBM-MT) did not show improvement in maintaining the quality of samples compared to the control sample (BMBM-O). After 12 months storage, BMBM-MT diets had significantly higher intensity in oxidized oil aroma than BMBM-O. This was an unexpected result, as the study aimed at creating treatments of different oxidation levels, using natural and synthetic antioxidants. Changes in significantly different sensory characteristics (oxidized oil, rancid etc.) over storage time for BMBM samples were minimal and not necessarily directional. However, the effect of using antioxidants to preserve BMBM samples might be clearly seen if we kept samples for more than 12 months at ambient temperature. The retarded oxidation in the food by adding antioxidants did play an important role in maintaining sensory characteristics of the CBPM samples over a 12 month storage time. Certain aroma and flavor characteristics, especially oxidized oil and rancid, were clearly seen to increase for CBPM samples that were produced without antioxidant (CBPM-O). Further research is needed to determine the chemical compounds formed during the shelf-life of pet food diets manufactured with beef meat and bone meal to understand the oxidation process better.

Based on descriptive sensory analysis results, extruded pet foods prepared from BMBM had higher capability to maintain their sensory characteristics than pet foods prepared from CBPM. BMBM contains more saturated fatty acid which permits it to be more stable against oxidation than many of other rendered protein meals [2]. The level of fat can also affect the overall amount of oxidation. For example, Gray [7] evaluated the amount of crude fat in BMBM and CBPM and reported that BMBM from two different producers had crude fat between 8.4%–11.9% and chicken byproduct meal from three different producers had higher crude fat ranging between 13.2%–16.6% [7]. This would support our results in which the CBPM seemed change more over time. The 14 month shelf life study of dry pet food by Lin et al. [12] also showed that dry pet food with added poultry fat oxidized quicker than the sample with added beef fat.

The consumer acceptance study showed that small changes in both aroma and flavor characteristics of BMBM-O samples over storage time might be too small for pet owners to detect the differences and this resulted in no significant differences in all liking scores. On the other hand, pet owners were able to detect the differences in sensory characteristics of CBPM-O samples over storage time. The changes for oxidized oil and rancid aroma intensity in CBPM-O samples were bigger, especially when samples were kept for 12 months. These changes were large enough for consumers to detect the differences between samples and resulted in significant decreases in overall and aroma liking scores. This result showed that the higher intensity of rancidity-related sensory attributes, which were the indicators of lipid oxidation, did affect pet owners’ acceptability of extruded pet foods from rendered protein meal. Pet owners gave lower acceptability scores for samples with high intensity of off-note characteristics. However, the intensity of these sensory characteristics (evaluated by a highly trained sensory panel) had to be high enough for pet owners to recognize or distinguish. Consumers or pet owners might have a lower sensitivity than panelists who have been trained and exposed repeatedly to the particular sensory characteristics [19]. Chapman et al. [20] also reported that consumers had a higher threshold than trained panelists to detect light-oxidized flavors in milk. While trained panelists could identify light-oxidized flavors in milk after the milk was exposed to the light for 15–30 min, consumers could detect oxidized flavors in milk after 54 min to 2 h of light exposure. 

Interestingly, drivers of liking for BMBM-O samples showed that pet owners liked samples with higher off-note characteristics. This may be related to consumer expectations—the consumers may have expected a commercial dog or cat food to have certain aroma characteristics that are clearly perceivable. Aroma liking scores slightly increased for BMBM-O samples over time points (Table 8). Pet owners might have had their expectation about aroma of samples too. When they evaluated the fresh sample that had a lower intensity of aroma than they may have expected, they tended to decrease their liking scores. Pet owners were more likely to give a little bit higher score for BMBM-O samples that had been stored for longer time, although these samples had been reported to have higher off note characteristics. The intensity of the off note characteristics might have been too low for them to recognize as a “bad” aroma, but might have been enough for them to say that the aroma of the samples is not too low for them. Therefore, pet owners may have given higher scores for BMBM-O samples that they thought to have a higher aroma overall. The driver of liking for CBPM-O samples showed the opposite direction that pet owners preferred CBPM-O samples with lower in undesirable sensory characteristics. This meant the intensity of off-note characteristics in CBPM-O samples were high enough for pet owners to recognize as an undesirable aroma and resulted in lower acceptability for CBPM-O with longer storage time. The results from the current study are intriguing and warrant further research on the levels of aromatics of pet foods pet owners can distinguish and categorize as pleasant or unpleasant.

The extruded pet foods in this study were not coated with any flavors or fats when exiting the dryer in order to eliminate confounding factors aside from rendered protein meal, antioxidants, and storage time. Therefore, the changes in sensory properties and liking score overtime were based on factors of interest only. Some might argue that this approach would not be representative of commercial products, the intent was to focus on the sensory perception of oxidized meal impact. It is clear the technique did just that. Appearance seemed to be another factor that affected pet owners’ acceptability of extruded pet food. For the fresh samples, CBPM-O samples with more appealing appearance (no hair, white pieces) had higher acceptability scores than BMBM-O samples. However, the changes in acceptability scores over time for each diet shouldn’t be affected by appearance since samples from all storage time points had similar appearance. The significant decrease in liking scores over time was mainly due to the noticeable increase in undesirable aroma and flavor intensities. Di Donfrancesco et al. [16] reported that pet owners’ acceptability of dry pet food was more influenced by appearance than aroma of the products, however, too intense undesirable aroma was likely to affect consumer acceptance negatively.

Sensory analysis results showed that diets manufactured with CPBM-O had higher intensities in oxidized oil and rancid attributes after 12 month of storage which consumer could detect and considered as undesirable characteristics of the product. This resulted in a significant decrease in acceptability scores. Since pet food owners are the ones who make decision on purchasing food for their pets, measuring and setting acceptable level of oxidation by using human sensory analysis along with chemical analysis may be more appropriate method rather than depending on chemical characteristics of the products only.

## 5. Conclusions

Descriptive sensory analysis detected significant changes in pet food aroma and flavor characteristics for both the beef meal samples and the chicken byproduct meal samples. However, for chicken byproduct meal samples the differences were more pronounced and directional. This indicates that oxidation processes are not necessarily unidirectional and that further research is needed in the area of oxidation processes in pet foods manufactured with rendered meals. Lack of use of antioxidant, e.g., the high level of oxidation in CBPM samples influenced liking; but, this was not seen to the same magnitude in BMBM samples.

The consumer study showed no differences in consumer liking for beef meat and bone meal samples. This may have been caused by the low levels of aromatics of the samples. On the other hand, the noticeable increase in aroma characteristics in chicken byproduct meal samples over storage time had an effect on consumer liking. Consumers tended to give lower liking score for samples with either too low or too intense an aroma, but too intense aromas had more of a negative impact on sample liking.

## Figures and Tables

**Table 1:** Pet food diets produced with beef meat and bone meal (BMBM) and chicken byproduct meal (CBPM) oxidized to various levels.

Ingredient	BMBM Diet, % ***	CBPM Diet, %
Beef meat and bone meal	51.37	-
Chicken byproduct meal	-	37.80
Rice, Brewers	14.38	18.92
Corn	14.38	18.92
Wheat	14.38	18.92
Beet Pulp	4.00	4.00
Potassium Chloride	0.40	0.40
Monosodium Phosphate	0.25	-
Salt	0.25	0.25
Choline Chloride, 60% dry	0.20	0.20
Vitamin Premix *	0.15	0.15
Trace Mineral Premix **	0.10	0.10
DL Methionine	-	0.10
Taurine	0.05	-
**Nutrient Composition (as-is)**		
Moisture	10.00	10.00
Crude Protein	30.00	30.00
Crude Fat	5.81	6.58
Ash	16.56	7.45
Crude Fiber	2.66	2.56

* vitamin premix supplied calcium carbonate, zinc sulfate, ferrous sulfate, copper sulfate, manganous oxide, sodium selenite, calcium iodate. ** trace mineral premix supplied calcium carbonate, vitamin E supplement, niacin supplement, calcium pantothenate, vitamin A supplement, thiamine mononitrate, pyridoxine hydrochloride, riboflavin supplement, vitamin D3 supplement, biotin, vitamin B12 supplement, folic acid. *** Calculated values.

**Table 2:** Attributes, definitions, and references for descriptive analysis of extruded pet foods from rendered protein meals.

Attribute	Definition	Reference
Oxidized oil	The aromatic associated with aged or highly used oil and fat.	Microwave Oven Heated Wesson vegetable oil = 6.0 (aroma), 6.0 (flavor). Preparation: Add 300 mL of oil from a newly purchased and opened bottle of Wesson vegetable oil to a 1000 mL glass beaker. Heat in the microwave oven on high power for 3 min. Remove from microwave and let sit at room temperature to cool for approximately 25 min. Then heat another 3 min, let cool another 25 min, and heat for one additional 3 min interval. Let beaker sit on counter uncovered overnight. Serve 1 tablespoon of the oil in a medium snifter, covered with a watch glass (aroma) and pour in a 30 mL cup (flavor).
Stale	The aromatic impression that is flat, dull and lacks freshness.	Mission tortilla white flour = 2.0 (aroma), 2.0 (flavor). Preparation: Serve 4 pieces of 1” square in a medium snifter (aroma), and a 100 mL cup (flavor).
Cardboard	The aromatic associated with cardboard or paper packaging. The intensity rating is only for the “cardboardy” character within the reference.	Mission tortilla white flour = 2.5 (aroma). Preparation: Serve 4 pieces of 1” square in a medium snifter and covered with a watch glass (aroma). Mama Mary’s Pizza Crust = 3.0 (flavor). Preparation: Serve 1 piece of 2” square in a 100 mL cup (flavor) Cardboard = 7.5 (aroma). Preparation: 2” cardboard square in ½ cup of water. Serve in a medium snifter and covered with a watch glass (aroma).
Rancid	A somewhat heavy aromatic characteristic of old, oxidized, decomposing fat and oil. The aromatics may include painty, varnish, or fishy.	Microwaved Wesson vegetable oil (4 min at high) = 2.5 (aroma), 3.0 (flavor). Preparation: microwave 1 ½ cups oil on high power for 4 min. Let cool and Serve ¼ cup in a snifter and covered with a watch glass (aroma), pour in a 30 mL cup (flavor). Microwaved Wesson vegetable oil (5 min at high) = 5.0 (aroma), 5.0 (flavor). Preparation: microwave 1 ½ cups oil on high power for 5 min. Let cool and Serve ¼ cup in a snifter and covered with a watch glass (aroma), pour in a 30 mL cup (flavor).
Sour	The fundamental taste factor associated with a citric acid solution.	0.015% Citric Acid Solution = 1.5 0.050% Citric Acid Solution = 2.5
Bitter	The fundamental taste factor associated with a caffeine solution.	0.01% Caffeine Solution = 2.0 0.02% Caffeine Solution = 3.5 0.035 % Caffeine Solution = 5.0
Metallic	An aromatic and mouth feel associated with tin cans or aluminum foil.	0.10% Potassium Chloride Solution = 1.5

**Table 3:** Mean intensity scores **^1^** ± standard error (SEM) of aroma attributes for BMBM samples.

Aroma Attribute	Storage Time (Month)	Antioxidant Treatment			*p*-Level ^2^		
		BMBM-O	BMBM-MT	BMBM-ET	Antioxidant (A)	Time (T)	A × T
Oxidized oil (*n* = 12)	0	2.29 ^BC,3^ ± 0.10	2.29 ^C^ ± 0.07	2.33 ^C^ ± 0.09	0.0211	<0.0001	0.0329
	3	2.42 ^AB,ab,4^ ± 0.15	2.29 ^C,b^ ± 0.10	2.67 ^AB,a^ ± 0.07			
	6	2.00 ^C^ ± 0.19	2.21 ^C^ ± 0.07	2.25 ^C^ ± 0.12			
	9	2.46 ^AB^ ± 0.13	2.75 ^B^ ± 0.10	2.50 ^BC^ ± 0.06			
	12	2.63 ^A,b^ ± 0.13	3.21 ^A,a^ ± 0.18	2.83 ^A,b^ ± 0.07			
Stale (*n* = 12)	0	2.04 ^B,b^ ± 0.11	2.13 ^B,b^ ± 0.09	2.42 ^B,a^ ± 0.06	<0.0001	0.0008	0.0044
	3	2.25 ^AB,b^ ± 0.10	2.29 ^AB,b^ ± 0.07	2.88 ^A,a^ ± 0.13			
	6	2.04 ^B,b^ ± 0.20	2.29 ^AB,ab^ ± 0.10	2.46 ^B,a^ ± 0.11			
	9	2.38 ^A^ ± 0.07	2.42 ^A^ ± 0.10	2.33 ^B^ ± 0.07			
	12	2.42 ^A^ ± 0.08	2.54 ^A^ ± 0.10	2.46 ^B^ ± 0.07			
Cardboard (*n* = 12)	0	2.50 ± 0.06	2.54 ^B^ ± 0.04	2.71 ^B^ ± 0.07	0.1404	0.0301	0.0136
	3	2.58 ± 0.08	2.71 ^AB^ ± 0.07	3.04 ^A^ ± 0.13			
	6	2.58 ± 0.14	2.67 ^AB^ ± 0.11	2.50 ^B^ ± 0.11			
	9	2.67 ± 0.13	2.71 ^AB^ ± 0.10	2.67 ^B^ ± 0.11			
	12	2.67 ± 0.07	2.79 ^A^ ± 0.10	2.58 ^B^ ± 0.06			
Rancid (*n* = 12)	0	0.46 ^BC^ ± 0.17	0.17 ^C^ ± 0.11	0.08 ^C^ ± 0.08	0.9786	<0.0001	0.0004
	3	0.08 ^C^ ± 0.08	0.17 ^C^ ± 0.11	0.67 ^B^ ± 0.22			
	6	0.33 ^BC^ ± 0.14	0.17 ^C^ ± 0.11	0.00 ^C^ ± 0.00			
	9	1.04 ^A^ ± 0.25	0.92 ^B^ ± 0.24	0.79 ^B^ ± 0.23			
	12	0.71 ^BC^ ± 0.22	1.63 ^A^ ± 0.31	1.33 ^A^ ± 0.20			

**^1^** Scores are based on a 0–15-point numeric scale with 0.5 increments (0 = none and 15 = extremely high). Each mean score intensity is calculated from six panelists with two replicates (*n* = 12); **^2^** Probability of significant effects due to experimental oxidation treatment (A), storage time (T), and interaction effects (A × T); **^3^** Average for each parameter with different upper case letters (A–C) in the same column are significantly different (*p* ≤ 0.05) between times within treatment; **^4^** Averages with different lower case letters (a and b) in the same row are significantly different (*p* ≤ 0.05) between antioxidant treatments with respect to time.

**Table 4:** Mean intensity scores **^1^** ± standard error (SEM) of flavor attributes for BMBM samples.

Aroma Attribute	Storage Time (Month)	Antioxidant Treatment			*p*-Level ^2^		
		BMBM-O	BMBM-MT	BMBM-ET	Antioxidant (A)	Time (T)	A × T
Oxidized oil (*n* = 12)	0	2.50 ^B,3^ ± 0.09	2.46 ^B^ ± 0.10	2.42 ^B^ ± 0.08	0.6189	<0.0001	0.0164
	3	2.21 ^C^ ± 0.07	2.54 ^B^ ± 0.07	2.67 ^A^ ± 0.09			
	6	2.50 ^B^ ± 0.12	2.38 ^B^ ± 0.09	2.38 ^B^ ± 0.09			
	9	2.54 ^AB^ ± 0.10	2.42 ^B^ ± 0.08	2.54 ^AB^ ± 0.07			
	12	2.75 ^A^ ± 0.12	2.79 ^A^ ± 0.07	2.75 ^A^ ± 0.12			
Stale (*n* = 12)	0	2.42 ^BC^ ± 0.08	2.25 ^B^ ± 0.08	2.29 ^C^ ± 0.07	0.0362	0.0027	0.0394
	3	2.29 ^C,b,4^ ± 0.10	2.25 ^B,b^ ± 0.08	2.63 ^A,a^ ± 0.11			
	6	2.33 ^C^ ± 0.13	2.33 ^AB^ ± 0.07	2.38 ^BC^ ± 0.11			
	9	2.58 ^AB^ ± 0.06	2.38 ^AB^ ± 0.11	2.50 ^ABC^ ± 0.06			
	12	2.67 ^A^ ± 0.09	2.50 ^A^ ± 0.06	2.46 ^ABC^ ± 0.07			
Cardboard (*n* = 12)	0	2.67 ± 0.13	2.71 ± 0.07	2.79 ± 0.07	0.4397	0.3940	0.2088
	3	2.71 ± 0.10	2.83 ± 0.07	3.00 ± 0.12			
	6	2.63 ± 0.09	2.88 ± 0.11	2.67 ± 0.09			
	9	2.92 ± 0.06	2.75 ± 0.10	2.71 ± 0.07			
	12	2.67 ± 0.18	2.71 ± 0.11	2.79 ± 0.10			
Rancid (*n* = 12)	0	1.25 ^A^ ± 0.18	0.67 ^C^ ± 0.18	0.21 ^B^ ± 0.14	0.4879	<0.0001	<0.0001
	3	0.17 ^B^ ± 0.11	0.42 ^C^ ± 0.15	0.50 ^B^ ± 0.23			
	6	1.25 ^A^ ± 0.13	0.38 ^C^ ± 0.16	0.38 ^B^ ± 0.16			
	9	1.04 ^A^ ± 0.25	1.13 ^B^ ± 0.27	1.67 ^A^ ± 0.14			
	12	1.21 ^A^ ± 0.23	1.83 ^A^ ± 0.17	1.63 ^A^ ± 0.11			
Sour (*n* = 12)	0	1.54 ± 0.11	1.54 ± 0.13	1.50 ± 0.18	0.5611	0.6736	0.2243
	3	1.46 ± 0.10	1.29 ± 0.21	1.54 ± 0.17			
	6	1.50 ± 0.12	1.46 ± 0.14	1.38 ± 0.19			
	9	1.54 ± 0.11	1.38 ± 0.16	1.63 ± 0.11			
	12	1.58 ± 0.12	1.63 ± 0.09	1.33 ± 0.26			
Bitter (*n* = 12)	0	2.92 ± 0.20	2.79 ± 0.23	2.88 ± 0.29	0.1470	0.6056	0.8271
	3	2.88 ± 0.25	2.96 ± 0.24	2.83 ± 0.26			
	6	2.92 ± 0.24	2.83 ± 0.25	2.88 ± 0.28			
	9	2.92 ± 0.24	2.79 ± 0.25	2.75 ± 0.27			
	12	2.92 ± 0.24	2.71 ± 0.26	2.75 ± 0.26			
Metallic (*n* = 12)	0	1.17 ^ab^ ± 0.07	1.04 ^b^ ± 0.11	1.38 ^a^ ± 0.09	0.0412	0.1289	0.0032
	3	1.38 ^a^ ± 0.09	0.88 ^b^ ± 0.20	1.29 ^a^ ± 0.07			
	6	1.17 ± 0.13	0.92 ± 0.17	1.04 ± 0.17			
	9	1.17 ± 0.13	1.17 ± 0.13	1.29 ± 0.07			
	12	1.38 ^a^ ± 0.09	1.42 ^a^ ± 0.08	1.00 ^b^ ± 0.18			

**^1^** Scores are based on a 0–15-point numeric scale with 0.5 increments (0 = none and 15 = extremely high). Each mean score intensity is calculated from 6 panelists with two replicates (*n* = 12); **^2^** Probability of significant effects due to experimental oxidation treatment (A), storage time (T), and interaction effects (A × T); **^3^** Average for each parameter with different upper case letters (A–C) in the same column are significantly different (*p* ≤ 0.05) between times within treatment; **^4^** Averages with different lower case letters (a and b) in the same row are significantly different (*p* ≤ 0.05) between antioxidant treatments with respect to time.

**Table 5:** Mean intensity scores **^1^** ± standard error (SEM) of aroma attributes for CBPM samples.

Aroma Attribute	Storage Time (Month)	Antioxidant Treatment			*p*-Level ^2^		
		CBPM-O	CBPM-MT	CBPM-ET	Antioxidant (A)	Time (T)	A × T
Oxidized oil (*n* = 12)	0	2.29 ^C,3^ ± 0.10	2.38 ± 0.14	2.25 ± 0.14	<0.0001	<0.0001	<0.0001
	3	2.50 ^BC^ ± 0.06	2.38 ± 0.09	2.13 ± 0.09			
	6	2.88 ^B,a,4^ ± 0.22	2.46 ^b^ ± 0.17	2.33 ^b^ ± 0.07			
	9	2.50 ^BC^ ± 0.11	2.38 ± 0.13	2.13 ± 0.11			
	12	4.13 ^A,a^ ± 0.29	2.50 ^b^ ± 0.14	2.17 ^b^ ± 0.15			
Stale (*n* = 12)	0	2.38 ^B,a^ ± 0.11	2.25 ^AB,ab^ ± 0.10	2.00 ^B,b^ ± 0.11	<0.0001	0.0460	0.0528
	3	2.33 ^B^ ± 0.07	2.46 ^A^ ± 0.07	2.29 ^A^ ± 0.11			
	6	2.50 ^AB^ ± 0.11	2.33 ^AB^ ± 0.09	2.25 ^AB^ ± 0.08			
	9	2.33 ^B^ ± 0.17	2.17 ^B^ ± 0.09	2.17 ^AB^ ± 0.07			
	12	2.75 ^A,a^ ± 0.10	2.21 ^AB,b^ ± 0.10	2.21 ^AB,b^ ± 0.11			
Cardboard (*n* = 12)	0	2.75 ^a^ ± 0.16	2.54 ^ab^ ± 0.07	2.33 ^b^ ± 0.07	0.0014	0.7095	0.0739
	3	2.54 ± 0.10	2.46 ± 0.10	2.38 ± 0.09			
	6	2.54 ± 0.17	2.54 ± 0.10	2.50 ± 0.12			
	9	2.58 ± 0.12	2.50 ± 0.11	2.63 ± 0.13			
	12	2.83 ^a^ ± 0.09	2.42 ^b^ ± 0.08	2.21 ^b^ ± 0.10			
Rancid (*n* = 12)	0	0.33 ^C^ ± 0.19	0.63 ± 0.25	0.46 ± 0.25	<0.0001	<0.0001	<0.0001
	3	0.33 ^C^ ± 0.18	0.33 ± 0.18	0.58 ± 0.25			
	6	1.17 ^B^ ± 0.36	0.83 ± 0.22	0.46 ± 0.20			
	9	1.00 ^B^ ± 0.27	0.29 ± 0.22	0.50 ± 0.22			
	12	4.21 ^A,a^ ± 0.55	0.71 ^b^ ± 0.22	0.88 ^b^ ± 0.27			

**^1^** Scores are based on a 0–15-point numeric scale with 0.5 increments (0 = none and 15 = extremely high). Each mean score intensity is calculated from six panelists with two replicates (*n* = 12); **^2^** Probability of significant effects due to experimental oxidation treatment (A), storage time (T), and interaction effects (A × T); **^3^** Average for each parameter with different upper case letters (A–C) in the same column are significantly different (*p* ≤ 0.05) between times within treatment; **^4^** Averages with different lower case letters (a and b) in the same row are significantly different (*p* ≤ 0.05) between antioxidant treatments with respect to time.

**Table 6:** Mean intensity scores **^1^** ± standard error (SEM) of flavor attributes for CBPM samples.

Aroma Attribute	Storage Time (Month)	Antioxidant Treatment			*p*-Level ^2^		
		CBPM-O	CBPM-MT	CBPM-ET	Antioxidant (A)	Time (T)	A × T
Oxidized oil (*n* = 12)	0	2.29 ^C,3^ ± 0.13	2.50 ± 0.14	2.17 ^B^ ± 0.24	<0.0001	<0.0001	<0.0001
	3	2.54 ^C^ ± 0.13	2.42 ± 0.12	2.63 ^A^ ± 0.11			
	6	3.08 ^B^ ± 0.14	2.75 ± 0.16	2.42 ^AB^ ± 0.18			
	9	2.71 ^BC^ ± 0.14	2.50 ± 0.09	2.29 ^AB^ ± 0.10			
	12	3.96 ^A,a,4^ ± 0.26	2.75 ^b^ ± 0.18	2.46 ^AB,b^ ± 0.13			
Stale (*n* = 12)	0	2.33 ^B^ ± 0.09	2.38 ± 0.09	2.13 ± 0.13	0.0002	0.0338	0.1943
	3	2.29 ^B^ ± 0.10	2.38 ± 0.14	2.25 ± 0.10			
	6	2.42 ^B^ ± 0.10	2.50 ± 0.09	2.25 ± 0.08			
	9	2.50 ^B^ ± 0.12	2.46 ± 0.11	2.29 ± 0.10			
	12	2.83 ^A,a^ ± 0.17	2.50 ^b^ ± 0.09	2.21 ^c^ ± 0.10			
Cardboard (*n* = 12)	0	2.83 ^A,a^ ± 0.11	2.63 ^B,ab^ ± 0.09	2.38 ^B,b^ ± 0.09	0.0442	0.0046	0.1148
	3	2.50 ^B^ ± 0.11	2.63 ^B^ ± 0.13	2.54 ^AB^ ± 0.11			
	6	2.75 ^AB^ ± 0.12	2.96 ^A^ ± 0.10	2.75 ^A^ ± 0.08			
	9	2.96 ^A,a^ ± 0.11	2.67 ^AB,b^ ± 0.11	2.75 ^A,ab^ ± 0.12			
	12	2.83^A^ ± 0.09	2.75 ^AB^ ± 0.08	2.67 ^A^ ± 0.14			
Rancid (*n* = 12)	0	1.04 ^C,b^ ± 0.19	1.88 ^A,a^ ± 0.15	0.79B ^BC,b^ ± 0.25	<0.0001	<0.0001	<0.0001
	3	1.58 ^C^ ± 0.27	1.17 ^B^ ± 0.30	1.25 ^AB^ ± 0.29			
	6	2.83 ^B^ ± 0.23	1.92 ^A^ ± 0.10	1.00 ^ABC^ ± 0.29			
	9	1.17 ^C^ ± 0.27	1.08 ^B^ ± 0.31	0.50 ^C^ ± 0.21			
	12	3.92 ^A,a^ ± 0.20	1.13 ^B,b^ ± 0.31	1.50 ^A,b^ ± 0.31			
Sour (*n* = 12)	0	1.63 ± 0.13	1.75 ± 0.13	1.58 ± 0.17	0.1632	0.1806	0.7205
	3	1.67 ± 0.13	1.54 ± 0.16	1.67 ± 0.15			
	6	1.75 ± 0.17	1.79 ± 0.13	1.67 ± 0.13			
	9	1.67 ± 0.20	1.58 ± 0.12	1.38 ± 0.19			
	12	1.67 ± 0.15	1.58 ± 0.12	1.50 ± 0.22			
Bitter (*n* = 12)	0	2.92 ± 0.24	2.92 ^AB^ ± 0.29	3.00 ± 0.27	0.0556	0.0192	0.1458
	3	2.92 ± 0.27	2.67 ^B^ ± 0.30	2.88 ± 0.27			
	6	3.17 ± 0.25	3.13 ^A^ ± 0.20	2.92 ± 0.29			
	9	3.21 ± 0.23	2.83 ^AB^ ± 0.32	3.08 ± 0.29			
	12	3.21 ± 0.28	3.13 ^A^ ± 0.25	2.88 ± 0.23			
Metallic (*n* = 12)	0	0.92 ^B^ ± 0.20	1.00 ^AB^ ± 0.15	1.13 ^AB^ ± 0.16	0.5335	0.0055	0.3876
	3	1.08A ^B^ ± 0.17	0.83 ^B^ ± 0.20	0.75 ^B^ ± 0.20			
	6	1.04A ^B^ ± 0.16	1.25 ^A^ ± 0.14	1.21 ^A^ ± 0.13			
	9	1.25 ^AB^ ± 0.16	1.00 ^AB^ ± 0.16	0.88 ^AB^ ± 0.20			
	12	1.38 ^A^ ± 0.15	1.33 ^A^ ± 0.07	1.21 ^A^ ± 0.13			

**^1^** Scores are based on a 0–15-point numeric scale with 0.5 increments (0 = none and 15 = extremely high). Each mean score intensity is calculated from 6 panelists with 2 replicates (*n* = 12); **^2^** Probability of significant effects due to experimental oxidation treatment (A), storage time (T), and interaction effects (A × T); **^3^** Average for each parameter with different upper case letters (A–C) in the same column are significantly different (*p* ≤ 0.05) between times within treatment; **^4^** Averages with different lower case letters (a–c) in the same row are significantly different (*p* ≤ 0.05) between antioxidant treatments within time.

**Table 7:** Demographic information, storage and feeding behavior of participants in the consumer study.

**Demographic Information**		**Consumer (%)**
Gender	Female	69.8%
	Male	30.2%
Age	18–24	9.4%
	25–34	18.9%
	35–44	14.2%
	45–54	24.5%
	55–64	29.2%
	65 or older	3.8%
Education	Some school but no degree	1.9%
	High school degree	2.8%
	Some college but no degree	18.9%
	College degree	46.2%
	Graduate/Professional school degree	30.2%
Household Income (USD)	Less than 25,000	5.7%
	25,000–50,000	29.2%
	51,000–100,000	47.2%
	Over 100,000	17.9%
Pet owner	Dog	58.4
	Cat	20.8
	Both Dog and Cat	20.8
Dog’s breed type	Purebred	50.0
	Mixed Breed	41.7
	Both	8.3
Cat’s breed type	Purebred	4.5
	Mixed Breed	91.0
	Both	4.5
**Storage and Feeding Behavior**		**Consumer (%)**
Method for storing dry pet food	Airtight containers	49.1%
	In the original packaging LEFT OPEN to the air	13.2%
	In the original packaging RESEALED	34.9%
	Other	2.8%
Time for finishing a package of dry pet food	1 month	68.9%
	1 week	13.2%
	3 months	16.0%
	6 months	1.9%
Money spending on pet food per month	Less than $100	86.8%
	$100–$300	13.2%

**Table 8:** The mean scores **^1^** ± standard error (SEM) for overall liking, appearance liking, and aroma liking for BMBM-O **^2^** and CBPM-O **^3^** samples for each storage time point (*n* = 106).

Sample	Time Point (Month)	Overall Liking	Appearance Liking	Aroma Liking
**BMBM-O**	0	4.97 ± 0.18	4.66 ± 0.18	5.08 ± 0.18
	3	4.81 ± 0.18	4.47 ± 0.18	5.05 ± 0.17
	6	4.73 ± 0.19	4.61 ± 0.18	4.99 ± 0.17
	9	4.87 ± 0.18	4.67 ± 0.18	5.22 ± 0.18
	12	5.13 ± 0.18	4.92 ± 0.19	5.29 ± 0.17
	*p*-level **^4^**	0.5227	0.4398	0.7108
**CBPM-O**	0	5.52 ^a,5^ ± 0.16	5.42 ± 0.15	5.53 ^a^ ± 0.17
	3	5.39 ^a^ ± 0.16	5.23 ± 0.17	5.35 ^ab^ ± 0.16
	6	5.32 ^a^ ± 0.17	5.23 ± 0.16	5.22 ^ab^ ± 0.17
	9	5.24 ^a^ ± 0.18	5.25 ± 0.16	5.01 ^bc^ ± 0.19
	12	4.95 ^b^ ± 0.17	5.05 ± 0.15	4.79 ^c^ ± 0.20
	*p*-level **^2^**	0.0013	0.0797	0.0004

**^1^** Scores are based on a 9-point hedonic scale (1 = dislike extremely, 9 = like extremely); **^2^** Unpreserved beef meat and bone meal diet; **^3^** Unpreserved chicken byproduct meal diet; **^4^** Probability of significant effect due to storage time; **^5^** Averages with different lower case letters (a–c) indicate significant difference between storage time points of each diet and liking attribute (*p* ≤ 0.05).

**Table 9:** Mean drop in aroma liking associated with aroma attribute for BMBM-O **^1^** and CBPM-O **^2^** samples (*n* = 106).

Sample	Level	Frequencies	Consumer (%)	Mean Drops	*p*-Value ^4^
**BMBM-O**	Too little	44	41.51%	0.685	**0.048**
	JAR **^3^**	45	42.45%		
	Too much	17	16.04%	2.139	
**CBPM-O**	Too little	28	26.42%	0.536	0.240
	JAR	56	52.83%		
	Too much	22	20.75%	1.166	**0.020**

**^1^** Unpreserved beef meat and bone meal diet; **^2^** Unpreserved chicken byproduct meal diet; **^3^** JAR—Just About Right; **^4^** *p*-value considered significant at *p* < 0.05.
